# Monthly Summary

**Published:** 1876-01

**Authors:** 


### MONTHLY SUMMARY.

---

*Croton Chloral Hydrate in Neuralgia.*—Dr. Spender, in the *British Medical Journal*, says:—The power of this medicine over neuralgia seems so unquestionable, that I am confident it will soon occupy a leading place among our antidotes to pain. There are certain cases of what Dr. B. W. Richardson calls "neuralgic fever," *i. e.* general neuralgia with some pyrexia, which are completely controlled and ultimately cured by a combination of quinine and croton-chloral. In an instance of this sort very lately under my care, a maiden female, aged 49, I gave a grain of quinine in solution with three grains of croton chloral, in a pill, every two hours during the daytime; and, when the desired success was obtained, the medicines were gredually left off. Moreover, there is this good point about chroton-chloral, that, even if it fail to produce any benefit, it never seems to do any harm.—*Med & Surg. Reporter.*

